# Interlimb Coordination Performance in Seated Position in Persons With Multiple Sclerosis: Reduced Amplitude Over 6 min and Higher Coordination Variability in Persons With Walking Fatigability

**DOI:** 10.3389/fnhum.2021.765254

**Published:** 2021-10-20

**Authors:** Mieke Goetschalckx, Fanny Van Geel, Raf Meesen, Lisa Tedesco Triccas, Marc Geraerts, Lousin Moumdjian, Peter Feys

**Affiliations:** ^1^REVAL Rehabilitation Research Center, Faculty of Rehabilitation Sciences, Hasselt University, Hasselt, Belgium; ^2^Universitair Multiple Sclerosis Centrum (UMSC), Hasselt-Pelt, Hasselt, Belgium; ^3^Faculty of Art and Philosophy, IPEM, Institute of Psychoacoustic and Electronic Music, Ghent University, Ghent, Belgium

**Keywords:** multiple sclerosis, phase coordination index, seated coordination task, coordination, fatigability

## Abstract

**Background:** Walking fatigability is prevalent in MS and can be measured by a percentage distance decline during a 6-min walking test. Walking is characterized by an accurate and consistent interlimb antiphase coordination pattern. A decline in coordination each minute during a 6-min walking test is observed in persons with MS (pwMS). Measuring coordination during a 6-min seated coordination task with minimized balance and strength requirements, is assumed to examine a more fundamental interlimb antiphase coordination pattern in pwMS. This research aimed to answer the following research question: How does interlimb antiphase coordination pattern change during a seated coordination task in pwMS with walking fatigability (WF), non-walking fatigability (NWF) and Healthy Controls (HC)?

**Methods:** Thirty-five pwMS and 13 HC participated. Interlimb coordination was assessed by a seated 6-min coordination task (6MCT) with the instruction to perform antiphase lower leg movements as fast as possible. Outcomes were Phase Coordination Index (PCI) and movement parameters (amplitude, frequency).

**Results:** Mixed models revealed a significant effect of time for the the variability of generating interlimb movements, with a difference in mean values between WF and HC. A significant group^∗^time interaction effect was found for movement amplitude, represented by a significant decrease in movement amplitude in the WF group from minute 1 to the end of the task.

**Conclusion:** The higher variability in interlimb coordination and decrease in movement amplitude over time during the 6MCT in the WF group could be an indicator of decreased control of fundamental antiphase coordination pattern in pwMS with walking fatigability.

**Clinical Trial Registration:** www.clinicaltrials.gov, identifier NCT04142853 (registration date: October 29, 2019) and NCT03938558 (registration date: May 6, 2019).

## Introduction

Fatigue is highly reported in people with MS (pwMS), with a prevalence up to 80%, showing a high impact on quality of life (Kluger et al., 2013; Filippi et al., 2018). Recent taxonomies have indicated two types of fatigue: trait fatigue and state fatigue. Trait fatigue is referred to as an overall perception of fatigue over a longer time which is frequently assessed by questionnaires such as the modified fatigue impact scale (MFIS). State fatigue is activity dependent. State fatigue can be either assessed by asking the perceived fatigue by a visual analog scale (VAS), or assessed objectively by performance decline during, for example, maximal strength contractions or walking (Kluger et al., 2013; Van Geel et al., 2019). So far, research on motor fatigability has often been based on evaluation of the capacity to maintain maximal isometric muscle force measured on the body function level of International Classification of Functioning, disability and health (ICF); or the susceptibility to muscle fatigue (Severijns et al., 2017). With the use of the twitch interpolation technique, it has shown that motor fatigability in MS is mainly influenced by central factors such as reduced voluntary central drive. These theories assume a larger contribution of central than peripheral neural activation to motor fatigability (Steens et al., 2012; Bachinger et al., 2019; Severijns et al., 2019).

In recent years the focus of the assessment of motor fatigability have been changed from body function level of ICF to walking at ICF activity level. Walking-related fatigability has been quantified in pwMS by comparing walking velocity during different paradigms (Phan-Ba et al., 2012), and more recently, by comparing the last vs. the first minute of the 6 min walking test (6MWT), quantified by a “distance walked index” (Leone et al., 2016; Van Geel et al., 2020). Walking-related fatigability limits the mobility of pwMS in daily life, and is prevalent in up to half of pwMS with moderate to severe impairments (Leone et al., 2016). During the 6MWT, pwMS perceive more balance problems and gait impairments, and report overall higher state fatigue levels across minutes (Van Geel et al., 2021).

Walking requires accurate and consistent antiphase interlimb coordination pattern, to ensure safe ambulatory functions. Interlimb coordination involves a distributed network at both supraspinal and spinal level (Swinnen, 2002). It has been suggested that defaults in the central nervous system (Debaere et al., 2001; Swinnen, 2002; Swinnen and Carson, 2002), such as muscle firing timing, slowed motor nerve conduction time and reduced voluntary drive by the motor cortex (Swinnen and Carson, 2002; Wurdeman et al., 2011; Swanson and Fling, 2018) may contribute to the impaired coordination. When walking at self-selected and fast speed, pwMS with mild to moderate disability shows a worse interlimb coordination pattern than healthy controls (HC) (Richmond et al., 2020). Moreover, when performing a 6-min walking task, interlimb coordination pattern deteriorates each minute (Plotnik et al., 2020). Interlimb coordination in pwMS may be impacted by the presence of heterogenous symptoms, ranging from impaired dynamical balance, decreased muscle strength, asymmetric gait, compensatory movements, or perception of fatigue or performance fatigability. Therefore, a seated 6-min coordination task (6MCT) was designed to mimic the 6-min walking test (6MWT) but with minimalized balance requirements or major muscular effort, which are required during a regular walking task. Our main hypothesis is that a change in coordination pattern would be observed during the seated 6MCT, by the end of the task as a result of fatigability. This change is hypothesized to be more present in a group with walking-related performance fatigability, as both walking fatigability and coordination are assumed to be mostly (but not exclusively) influenced by central neural activation mechanisms.

The aim of this study was consequently to examine more fundamental antiphase interlimb coordination pattern while seated in pwMS, with and without walking fatigability in comparison to healthy controls.

## Materials and Methods

### Participants

The present study reports on exploratory observational baseline data of the clinical trials registered at www.clinicaltrials.gov: NCT04142853 (MS *n* = 27, HC *n* = 13) and NCT03938558 (MS *n* = 18). Seven pwMS participated in both studies and therefore, data of their first participation (NCT03938558) was included. PwMS and healthy controls (HC) were tested at the REVAL Rehabilitation Research Centre of Hasselt University, and recruited via REVAL, Rehabilitation, the MS Centre Overpelt and the non-for-profit organization Move To Sport. The ethical committee of Hasselt University, MS Centre Overpelt and Hospitalo-Facultaire Universitaire de Liège approved the protocols (B707201835771 and B9115201836892) and written informed consent was obtained from all participants. Participants were aged between 18 and 70 years, and had to be able to walk for 6 min with or without support (bilateral or unilateral). PwMS were diagnosed according to the McDonald criteria (Thompson et al., 2018). Three pwMS were excluded as they could not perform the 6MWT or the 6MCT safely.

### Descriptive Data

Age, gender, EDSS, Type MS, years since diagnosis, Timed 25 foot walk test (T25FW), Paced Auditory Serial Addition test (PASAT), Single Digit Modalities test (SDMT), Modified Fatigue Impact Scale (MFIS), and Multiple Sclerosis Walking Scale (MSWS-12), were collected in pwMS.

To divide patients into groups with or without walking fatigability, subjects performed the 6-min walking test (6MWT) according to the script of Goldman et al. (2008) in a 30-m corridor, where participants were asked to walk as fast as possible and cover as much distance as possible, making them challenge their maximal effort. Distance walked each minute was collected. The Distance Walked Index (DWI_6–1_) (Leone et al., 2016) was used to allocate pwMS into a walking fatigability (WF) and a non-walking fatigability group (NWF), according to the rounded cut-off value of −10% (Van Geel et al., 2020): [(distance walked in min 6 - distance walked in min 1)/distance walked in min 1]^∗^100. Before the start of the test (baseline), and after each minute of the 6MWT and 6-min coordination test (6MCT), participants were asked to rate their subjective perception of fatigue on a VAS scale, where 0 is no fatigue and 10 is extremely fatigued. Change in perceived fatigue was calculated as followed: (perceived fatigue VAS score in minute 6 - perceived fatigue VAS score at baseline) × 10.

### Apparatus Interlimb Coordination Test

**FIGURE 1:**
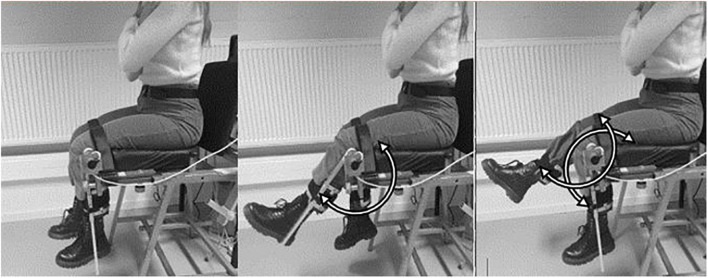
Representation of the seated antiphase interlimb coordination task. Subjects make simultaneous left and right knee flexion and extension antiphase movements.

To assess antiphase interlimb coordination of the lower limbs, participants performed a seated 6 min coordination task. Subjects were seated on a steel chair covered with foam, to which two levers were attached. To ensure a stable posture, subjects were strapped into the chair with a belt at the upper legs (Figure 1). The back was supported, but not fixed, so that participants could sit in the most comfortable position for them to perform the task. Participants were asked to keep their arm crossed by the shoulders throughout the 6MCT. The lower limbs were attached to levers to allow the lower legs to perform knee flexion and extension movements. The lateral joint line of the knee was aligned with the axis of rotation of the levers. At full knee extension, the mass of the each lever was 540 grams. Joint angles were registered by means of 2 incremental shaft encoders (Hengstler^®^, 1,000 bits per revolution, accuracy = 0.36°, sampling frequency = 100 Hz; Romsey, United Kingdom) mounted at the axis of rotation of each lever.

### Methodology Interlimb Coordination Test

**FIGURE 2:**
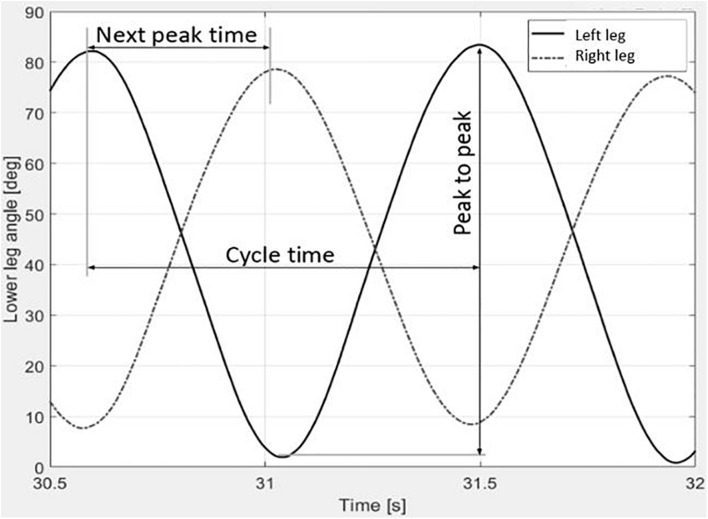
Visualization of movement cycles of left and right leg of a person with MS.

Participants were asked to perform flexion and extension movements at the knee joints in a coordinated antiphase pendulum movement. The movements had to be as fast or as many as possible for 6 min. For individualization purpose, no limits for range of motion were set. Participants performed antiphase interlimb movement for approximately 5 times, to ensure understanding and correct execution. The first 10 s of the test were excluded for data processing due to start-up movements. MATLAB (version R2019a) was used for data processing. An upsample rate of 10 was used for sample frequency. Figure 2 visualizes one movement cycle. Consequently, interlimb coordination parameters were analyzed and averaged per minute.

### Outcomes Interlimb Coordination Task

Originally, the phase coordination index (PCI) measures the consistency and accuracy in generating antiphase left-right leg movements while walking (Plotnik et al., 2007). The PCI was used to analyze interlimb coordination of the lower limbs during the seated 6MCT. Similar relative phase coordination outcomes were used previously, using a similar apparatus (Swinnen et al., 1997; Serrien, 1998; Serrien and Stelmach, 2000; Goetschalckx et al., 2021). Relative phase φ expressed the relative timing between the contralateral peaks [360^∗^(next peak time/cycle time)]. The accuracy in generating antiphase left-right leg movements is expressed by absolute error ABS(φ) = ((mean |φ*i*−180|)/180) × 100. The coefficient of variation (CV φ) was used to express the consistency in generating antiphase left-right leg movements CV(φ) = (Standard deviation φ/mean φ) × 100.

The PCI is the sum of the absolute error (ABSφ)and coefficient of variation (CV) of the relative phase (φ), expressed as a percentage. A lower PCI indicates a higher phase control and coordination.

The movement amplitude, seen as the spatial measure, consists of the peak-to-peak amplitude for each individual cycle (Serrien and Stelmach, 2000). Movement frequency, seen as the temporal measure, is expressed by the number of cycles per minute. One cycle was defined between two successive peak extension positions. The average movement amplitude and movement frequency for each leg were calculated for each minute.

### Data and Statistical Analysis

Data analysis was performed using SAS JMP^®^ Pro 14.10. (SAS Institute Inc., Cary, NC, United States). Linear mixed models were used to analyze the effect of group and time on interlimb coordination and spatiotemporal parameters, after checking the conditional residuals plots. To assess asymmetry between legs, mean values of movement amplitude and movement frequency of left and right leg were compared. Comparison of the mean values over 6MCT between left and right legs revealed no significant difference in any groups. Therefore, the analysis for spatiotemporal parameters during the 6MCT was performed with the mean value of both legs, per minute. Fixed effects were included in the mixed model which were group (WF, NWF, HC), time (minute 1, 2, 3, 4, 5, 6) and the interaction group^∗^time, using a Bonferroni correction for significance (*p* = 0.05/3). Participants were included as random factor. To compare factors between groups (HC, WF, NWF) a ANOVA was used, after normality was confirmed by Shapiro-Wilk test. For non-normal distributed data, the non-parametric Wilcoxon/Kruskal-Wallis test (Rank sums) was used. Two-sided *p*-values were set at alpha level of 0.05. If a significant effect existed, multiple comparison Tukey HSD was used for normal distributed data and Steel-Dwass method for non-normal distributed data. The Chi^2^ test was used to compare groups for nominal data.

## Results

### Demographics and Walking Fatigability

**FIGURE 3:**
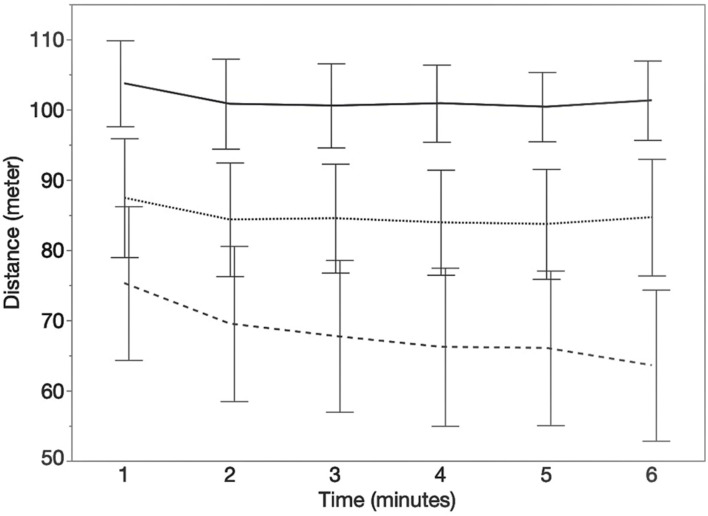
Course of the distance (meters) walked each minute during the 6MWT. Error bars represents 95% confidence intervals. Stripe pattern line represents persons with MS with walking fatigability (WF), dotted line represents persons with MS without walking fatigability (NWF) and solid line represents healthy controls (HC).

In total, 35 pwMS and 13 HC could be included in data analysis of 6MCT. Data of the 6MWT of one HC was excluded from descriptive analysis because the participant started to jog. In our study sample, 13 pwMS showed walking fatigability (WF = 37.15%). Figure 3 represents the distance walked each minute during the 6MWT (mean ± CI) for the different groups.

Descriptive and demographic outcomes are shown in Table 1. Significant differences between three groups were found for TF25W, DWI, 6MWT, and MSWS-12.

**TABLE 1:** Descriptive, demographic characteristics and interlimb coordination outcomes (mean, standard deviation) in HC and pwMS (WF-NWF).

	MS			HC	
		
	Total (*n* = 35)	WF (*n* = 13)	NWF (*n* = 22)	HC (*n* = 13)	*p*-value
**Descriptive variables**					
Age (years)					
Mean+SD 95%CI	48.9 ± 9.3 [45.8; 52.1]	49.8 ± 6.4 [45.9; 53.6]	48.5 ± 10.7 [43.7; 53.2]	51.7 ± 16.5 [41.7; 61.7]	NS^b^
Gender: female					
(n; %)	29; 82.9%	12; 92.3%	17; 77.3%	8; 61.5%	NS^c^
EDSS (0–10)					
Mean+SD 95%CI Missing data	3.3 ± 1.5 [2.7; 3.9] *n* = 9	3.7 ± 1.2 [2.9; 4.5] *n* = 3	3.0 ± 1.7 [2.1; 3.9] *n* = 6	NA	NS^a^
Type of MS (RR/PP/SP)					
Mean+SD Missing data	25/2/2 *n* = 6	10/0/0 *n* = 3	15/2/2 *n* = 3	NA	NS^c^
Years since diagnosis					
Mean+SD 95%CI Missing data	13.1 ± 6.0 [10.9; 15.3] *n* = 4	13.0 ± 6.4 [8.7; 17.3] *n* = 2	13.1 ± 5.9 [10.3; 15.9] *n* = 2	NA	NS^a^
**Motor outcomes**					
T25FW (s)					
Mean+SD 95%CI	5.1 ± 1.5 [4.5; 5.6]	5.4 ± 1.3 [4.7; 6.2]	4.9 ± 1.7 [4.1; 5.6]	3.3 ± 0.4 [3.1; 3.6]	**<0.0001^b^ ^WF–HC (0.0014)^ ^NWF–HC (< 0.0001)^**
Walking fatigability (DWI %)					
Mean+SD 95%CI Missing data	−8.0 ± 8.0 [−10.7; −5.2]	−16.2 ± 5.8 [−19.8; −12.7]	−3.1 ± 4.0 [−4.9; −1.3]	−2.3 ± 2.7 [−3.98; −0.5] *n* = 1	**<0.0001^a^ ^WF–HC (< 0.0001)^ ^WF–NWF (< 0.0001)^**
6MWT (m)					
Mean+SD 95%CI Missing data	474 ± 118 [433; 514]	414 ± 115 [345; 484]	509 ± 108 [461; 556]	608 ± 53 [574; 642] *n* = 1	**<0.0001^a^ ^WF–HC (< 0.0001)^ ^NWF–HC (0.0079)^ ^WF–NWF (0.0094)^**
**Cognitive outcomes**					
PASAT (./60)					
Mean+SD 95%CI	47 ± 9 [44; 50]	49 ± 7 [45; 54]	46 ± 10 [41; 50]	49 ± 9 [43; 55]	NS^a^
SDMT (./110)					
Mean+SD 95%CI	55 ± 13b [51; 60]	52 ± 13 [44; 60]	57 ± 13 [51; 63]	62 ± 12 [55; 70]	NS^a^
**Questionnaires**					
MFIS (./84)					
Mean+SD 95%CI Missing data	39 ± 16 [33; 45] *n* = 6	45 ± 12 [36; 55] *n* = 4	36 ± 16 [28; 43] *n* = 2	NA	NS^a^
MSWS-12 (./60)					
Mean+SD 95%CI Missing data	32 ± 14 [27; 38] *n* = 6	42 ± 8 [36; 49] *n* = 4	28 ± 14 [21; 34] *n* = 2	NA	**0.013^b^ ^WF–NWF^**
**Interlimb coordination outcomes (6MCT)**					
PCI					
Mean+SD 95%CI	15.9 ± 9.5 [12.6; 19.2]	17.7 ± 8.8 [12.4; 23.0]	14.8 ± 10 [10.4; 19.3]	10.3 ± 4.7 [7.4; 13.1]	**0.028^b^ ^WF–HC^**
Coefficient of variation (CV) φ					
Mean+SD 95%CI	8.1 ± 6.1 [6.0; 10.1]	8.6 ± 4.2 [6.0; 11.1]	7.8 ± 7.0 [4.7; 10.9]	5.1 ± 1.6 [4.1; 6.0]	**0.011^b^ ^WF–HC^**
Absolute error (ABS) φ					
Mean+SD 95%CI	7.8 ± 5.1 [6.1; 9.6]	9.1 ± 5.7 [5.6; 12.6]	7.1 ± 4.7 [5.0; 9.2]	5.2 ± 4.7 [2.4; 8.1]	NS^b^
Movement amplitude					
Mean+SD 95%CI	70.0 ± 21.9 [62.4; 77.4]	61.3 ± 21.1 [48.5; 74.0]	75.1 ± 21.1 [65.7; 84.4]	77.7 ± 24.9 [62.7; 92.8]	NS^a^
Movement frequency					
Mean+SD 95%CI	72.4 ± 13.7 [67.7; 77.1]	72.3 ± 16.0 [62.6; 81.9]	72.4 ± 12.5 [66.9; 78.0]	81.5 ± 22.7 [67.8; 95.2]	NS^b^

*MS, Multiple sclerosis; WF, walking fatigability group; NWF, non-walking fatigability group; HC, Healthy controls; EDSS, Expanded Disability Status Scale; RR, relapsing remitting MS; PP, primary progressive MS; SP, secondary progressive MS; T25FW, Timed 25-foot walk test; DWI, Distance walked index; 6MWT, 6 min walking test; PASAT, Paced auditory serial addition test; SDMT, Symbol digit modality test; MFIS, Modified fatigue impact scale; MSWS-12, Multiple sclerosis walking scale; PCI, Phase coordination index; ns, not significant; NA, not applicable.*

*Bold: significant effect p ≤ 0.05, NA, Not applicable, NS, not significant (p > 0.05), ^*a*^Independent t-test or ANOVA (post hoc Tukey HSD), ^*b*^Wilcoxon or Kruskal-Wallis test (post hoc using Steel-Dwass method), ^*c*^Chi^2^ test, n, Number, 95% CI, 95% Confidence interval [lower 95%; upper 95%], SD, Standard deviation.*

**FIGURE 4:**
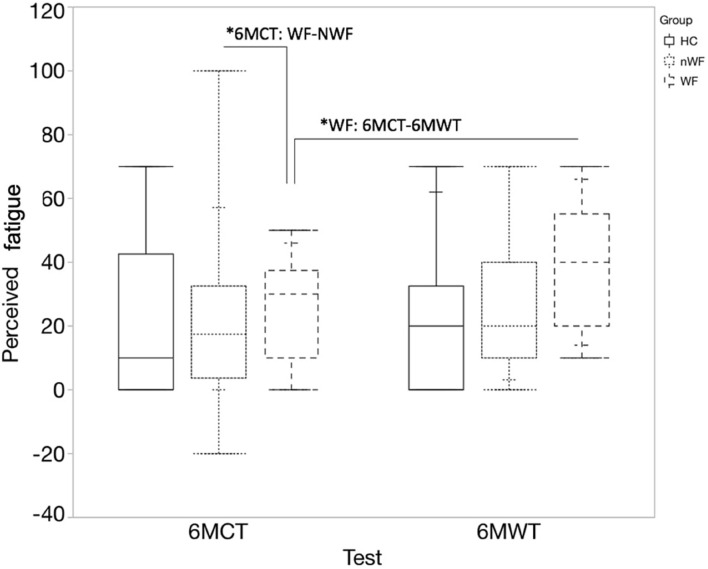
Median change in perceived fatigue (quartiles) during 6MWT and 6MCT. Full blocks represent data of healthy controls (HC); block with stripes pattern represents persons with MS with walking fatigability (WF), block with dot pattern represents persons with MS without walking fatigability (NWF). *6MCT: WF-NWF: significant difference in change in perceived fatigue between the WF and NWF group for the 6MCT (*p* = 0.042). *WF: 6MCT-6MWT: significant difference in change in perceived fatigue between 6MCT and 6MWT in persons with WF (*p* = 0.036).

Figure 4 represents the median (quartiles) of the change in perceived fatigue for each test and group. No significant differences were found between change in perceived fatigue during the 6MWT and 6MCT in HC and NWF group. However, in pwMS with WF, the 6MWT led to a significant higher change in perceived fatigue compared to the 6MCT [*t*(23.44) = 2.23; *p* = 0.036]. Additionally, a significant difference was found between WF-NWF (*MD* = 8.51; *SE* = 3.53; *z* = 2.41; *p* = 0.042) for the change in perceived fatigue for the 6MCT.

### Interlimb Coordination

**FIGURE 5:**
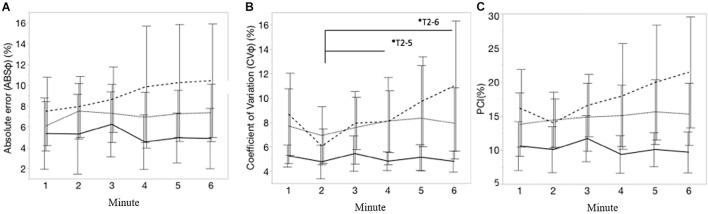
**(A–C)** Represents, respectively, the course of absolute error of the relative phase (ABSφ, %), coefficient of variation of relative phase (CV φ; %) and the phase coordination index (PCI, %), during each minute of the 6MCT. Error bars represents 95% confidence intervals. Stripe pattern line represents persons with MS with walking fatigability (WF), dotted line represents persons with MS without walking fatigability (NWF) and solid line represents healthy controls (HC). *T2–5, *T2–6: significant main effect of time for CV(φ) from minute 2 to 5 (*p* = 0.0161) and minute 2 to 6 (*p* < 0.01).

Mean values of all interlimb coordination outcomes measures are shown in Table 1. Figures 5A–C visualize, respectively, the course of the ABS φ, CV φ, and PCI during each minute of the 6MCT (mean ± CI). A main effect of time was observed for the CV φ (variability in generating antiphase left-right leg movements) [*F*(5, 225) = 3.20; *p* < 0.01]. After *post hoc* multiple comparison significant differences were seen in CV φ from minute 2–5 [*t*(225) = −3.26; *p* = 0.0161] and 2–6 [*t*(225) = −3.57; *p* < 0.01] (see Figure 5). Mixed model analysis revealed no significant main effect of time, group, nor an interaction effect of group^∗^time for PCI and ABS φ.

The mean values of most interlimb coordination parameters differed significantly between HC and PwMS with WF (PCI_mean_: *MD* = 7.69; *SE* = 3.00; *z* = 2.56; *p* = 0.028; CV_mean_: *MD* = 8.61; *SE* = 3.00; *z* = 2.87; *p* = 0.011). Supplementary Table 1 displays mean ± standard deviation (SD) of the interlimb coordination outcomes each minute.

**FIGURE 6:**
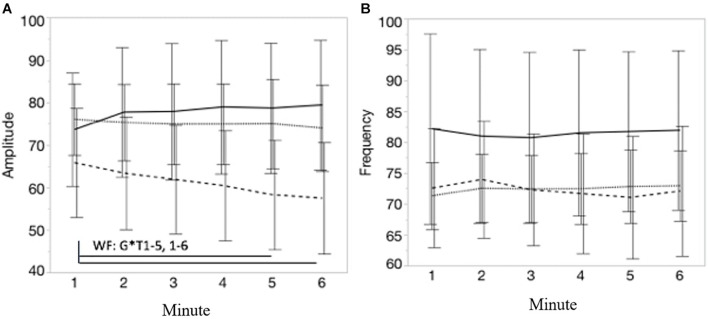
**(A,B)** Represents, respectively, the course of the movement amplitude and movement frequency during each minute of the 6MCT. Error bars represents 95% confidence intervals. Stripe pattern line represents persons with MS with walking fatigability (WF), dotted line represents persons with MS without walking fatigability (NWF) and solid line represents healthy controls (HC). *WF: G*T1–5, 1–6: significant interaction effect of group*time: pwMS with WF decreased their movement amplitude significantly from minute 1 to 5 (*p* < 0.01) and minute 6 (*p* < 0.00).

Figures 6A,B visualize, respectively, the course of the movement amplitude and movement frequency during each minute of the 6MCT (mean ± CI). A significant interaction effect of group^∗^time was found for movement amplitude [*F*(10, 225) = 4.57; *p* < 0.001]. *Post-hoc* multiple comparison showed that pwMS with WF decreased their movement amplitude significantly from minute 1 to both minute 5 [*t*(225) = 4.40; *p* < 0.01] and 6 [*t*(225) = 4.89; *p* < 0.001]. In contrast to the movement amplitude, no significant main time effect, nor an interaction effect was observed for movement frequency, suggesting that no group significantly changed their movement frequency over the 6MCT. Supplementary Table 2 displays the spatiotemporal movement parameters of each group by time (mean ± SD).

## Discussion

This is the first study that investigates an antiphase coordination pattern of the lower limbs during a seated 6 min coordination task (6MCT) in pwMS and changes over time. The seated 6MCT was designed to examine more fundamental interlimb coordination of the lower limbs, assuming minimized balance and strength demands, which are required during walking. Our main hypothesis was that a change in coordination pattern over time, due to performance fatigability, would be observed during seated 6MCT in pwMS with WF, by means of change in antiphase coordination movements (ABS, CV, PCI), and decrease in spatial and temporal movement parameters (movement amplitude, movement frequency). We hypothesized this change, as a similar trend of deterioration in coordination pattern over each minute have been observed in pwMS while performing a 6-min walking task (Plotnik et al., 2020).

The exploratory results of this study showed that interlimb coordination changes over time, observed by an increase in their variability in generating antiphase left-right movement CV(φ), toward the end of the task. As expected, this change in variability was greatest in pwMS with WF. Besides the observed change over time, the mean values of CV(φ) and PCI differed significantly between HC and pwMS with WF, with worse values in pwMS with WF. Noteworthy is that ABS(φ) in antiphase generation did not differ between group and did not changed significantly over time. These results suggest that pwMS with WF have more difficulties in the control of seated antiphase coordination of the lower limbs compared to HC.

Regarding the temporal and spatial movement parameters during the 6MCT, we expected to observe a similar trend of decline in performance in movement frequency or amplitude in pwMS with WF, as they show a decrease in walking distance during the 6MWT. As the 6MWT requires not only a consistent and accurate antiphase coordination pattern, but also balance and strength, the 6MCT was designed to minimalize balance requirements or major muscular effort, and therefore in theory stresses more central neural mechanism. This 6MCT was based on a similar test in previous research to measure phase coordination deficits, in stroke, elderly and Parkinson (Swinnen et al., 1997; Serrien, 1998; Serrien and Stelmach, 2000). We found that only in pwMS with WF, the movement amplitude significantly decreased over time, and not movement frequency. One of the first studies that used this interlimb seated coordination task in Parkinson disease found significant differences in movement amplitude compared to HC. Temporal measure (cycle duration) and absolute error (ABS) did not significantly differ between patients and HC. Together, these results suggest that during a seated coordination task, the temporal movement parameter is less influenced by neurological diseases, like pwMS and Parkinson disease, than spatial movement parameters. However, direct comparison is not possible due to difference in population, but also in length of the task (24 trials of 15 s vs. sustained 6 min) and performing the task while listening to a metronome (1 Hz) (Swinnen et al., 1997).

In the WF group, the change in perceived state fatigue during the 6MCT was lower than during the 6MWT, indicating that the seated task was perceived less fatiguing than walking. However, despite low levels of perceived state fatigue, the test detected differences in antiphase interlimb coordination pattern between groups. These results suggest that the 6MCT can be used as an alternative for patients who are not able to perform the 6MWT due to extreme fatigue or having severe motor disabilities. This assumption should, however, be further investigated, as the 6MCT cannot be directly compared to walking, as explained further. We note that, although pwMS without walking fatigability did show walking impairments assessed by the T25FWT and 6MWT, no significant differences were found in seated interlimb coordination performance, nor in change in perceived state fatigue, between pwMS in the NWF group and HC. Besides, we note that abnormal trait fatigue was present in many pwMS with WF (see Table 1, mean MFIS = 45), assessed by the MFIS. However, no significant difference in trait fatigue was found in pwMS with or without walking-related performance fatigability, and thus trait fatigue seems to be rather a general MS characteristic. This result may suggest that trait fatigue do not directly relates to walking-related performance fatigability. These results highlight the importance of interlimb coordination in walking-related performance fatigability.

In contrast to 6MCT, walking is for example characterized by different sensory-motor interactions, different and higher muscle activity, higher dynamic balance needs, metabolic cost, higher automaticity, etc., which all seems to be different in the 6MCT. Moreover, Meesen et al. (2005) found that movement with the head is a contributing factor for disturbance of coordination. Serrien and Stelmach (2000) confirms that movements integrating different limbs show more coordination effort in elderly. Future research could investigate these perturbations and multi-limb coordination patterns during the seated coordination test, to increase the ecologically validity of the 6MCT toward walking. The 6MCT should therefore be considered as a model that allows (in theory) isolation of the central neural drive mechanisms, taking lower limbs into account.

Future studies, should take the following limitation of this study into account. This research instructed participants to perform as many movements as possible, so move as fast as possible during 6 min, similar to the instructions of the 6MWT, where patients are instructed to walk as fast as possible, and cover as much distance as they can. Therefore, movement frequency could have been prioritized and the main focus of the participants over the 6 min instead of movement amplitude. The decrease in amplitude might have been a compensation strategy to keep the frequency equal, and perform as many movements as possible. Future studies could focus on different instructions and look at the possible influence of prioritization of amplitude or frequency. However, movement frequency is often prioritized in other tasks of the locomotor system, wherefore we do not think that the instruction has exclusively influenced our results (Malone et al., 2012; Motl et al., 2012; Comber et al., 2017).

Methodological considerations apply. The small sample size, especially in subgroups, and no *a priori* power analysis, should be considered when interpreting the results. A power analysis could not be made given it was the first time that the task was used in persons with MS. Besides, no one-to-one matching was performed between HC and MS groups. Next, we cannot exclude the impact of the relative weight of the levers, muscle strength asymmetries, accuracy of axis alignment of the device during the task, the liberty of comfortable range of motion of the legs and optional use of back support on differences in task performance between participants. Because of individualization purpose, participants were not restricted in their movement amplitude, nor externally paced in their movement frequency, similar to 6MWT were the step length and cadence are not standardized. One may argue that muscle strength asymmetries may have impacted the results. In this research, maximal lower limb strength was unfortunately not recorded given strength requirements in the seated coordination task were limited and likely not importantly affecting interlimb coordination control. Still, future research is advised to include maximal lower leg muscle strength as strength asymmetry might be present in pwMS (Farrell et al., 2021) and can then be included as factor in statistical analyses. Further research should also elaborate on more standardized task constraints regarding movement amplitude or correct for variability in movement amplitude to avoid that variability expressed in absolute values could influence group comparisons.

## Conclusion

Our exploratory results suggest that the antiphase coordination of the lower limbs change over time while performing a seated 6-min coordination task. These changes were apparent by an average higher coordination variability and a decrease in movement amplitude. The observed changes where more prominent in pwMS with walking-related performance fatigability. Results suggest indirectly the influence of central mechanism contributing to coordination patterns in the walking-related performance fatigability group. Further studies in a powered sample size with new methodological considerations are warranted to determine the relative contribution of centrally driven interlimb coordination and walking fatigability.

## Data Availability Statement

The raw data supporting the conclusions of this article will be made available by the authors after request.

## Ethics Statement

The studies involving human participants were reviewed and approved by the Ethical Committee of Hasselt University, MS Centre Overpelt and Hospitalo-Facultaire Universitaire de Liège approved the protocols (B707201835771 and B9115201836892). The patients/participants provided their written informed consent to participate in this study.

## Author Contributions

FV was responsible for study conceptualization, data collection, data processing, and writing of the manuscript. MGo was responsible for data analyzing, data interpretation, and writing of the manuscript. MGe was responsible for data processing. RM was responsible for equipment conceptualization, data processing, and data interpretation. LT and LM were responsible for data interpretation and writing of the manuscript. PF was responsible for study conceptualization, data interpretation, and writing of the manuscript. All authors contributed to the article and approved the submitted version.

## Conflict of Interest

PF was steering committee member of Neurocompass, participated to advisory board meetings of BIOGEN IDEC, and received teaching honoraria for EXCEMED and PARADIGMS. The remaining authors declare that the research was conducted in the absence of any commercial or financial relationships that could be construed as a potential conflict of interest.

## Publisher’s Note

All claims expressed in this article are solely those of the authors and do not necessarily represent those of their affiliated organizations, or those of the publisher, the editors and the reviewers. Any product that may be evaluated in this article, or claim that may be made by its manufacturer, is not guaranteed or endorsed by the publisher.
