# Supplementary material for: Interlimb Coordination Performance in Seated Position in Persons With Multiple Sclerosis: Reduced Amplitude Over 6 min and Higher Coordination Variability in Persons With Walking Fatigability
Source: Front Hum Neurosci. 2021 Oct 20;15:765254. doi: 10.3389/fnhum.2021.765254 (PMC8564500; doi:10.3389/fnhum.2021.765254)
Supplement: Supplementary file 1 [file Data_Sheet_1.docx]

**Interlimb coordination performance in seated position in persons with Multiple Sclerosis: reduced amplitude over six minutes and higher coordination variability in persons with walking fatigability**

Fanny Van Geel*^1,2^ PhD, Mieke Goetschalckx*^1^ MSc, Raf Meesen^1^ PhD, Lisa Tedesco Triccas^1^ PhD, Marc Geraerts^1^ Msc, Lousin Moumdjian ^1,2,3^ PhD, Peter Feys^1,2^ PhD

Affiliations:

^1^REVAL Rehabilitation Research Center, Faculty of Rehabilitation Sciences, Hasselt University, Hasselt, Belgium

^2^UMSC Hasselt-Pelt, Belgium

^3^ IPEM, Institute of Psychoacoustic and Electronic Music, Faculty of Art and Philosophy, Gent University, Gent, Belgium

**Corresponding author:**

Mieke Goetschalckx: mieke.goetschalckx@uhasselt.be, Agoralaan Gebouw A, 3590 Diepenbeek, Belgium, +32494748620

**Supplementary Table 1.** Interlimb coordination outcomes (mean±standard deviation) per group (HC-WF-NWF) by time (minute 1-6).

| **Outcome** | **Minute** | **Group** | | |
| --- | --- | --- | --- | --- |
|  |  | **WF (n=13)** | **NWF (n=22)** | **HC (n=13)** |
| **Absolute error (ABS)** $\boldsymbol{\varphi}$ | 1 | 7.49±5.44 | 6.07±5.32 | 5.35 ±5.66 |
|  | 2 | 7.94±4.84 | 7.52±6.02 | 5.30±6.32 |
|  | 3 | 8.64±5.21 | 7.29±6.29 | 6.25±5.19 |
|  | 4 | 9.83±9.67 | 6.93±5.42 | 4.55±4.34 |
|  | 5 | 10.23±9.30 | 7.26±5.11 | 4.95±3.97 |
|  | 6 | 10.44±9.01 | 7.36±6.24 | 4.89±4.80 |
| **Coefficient of variation (CV)** $\boldsymbol{\varphi}$ | 1 | 8.68±5.57 | 7.70±6.89 | 5.27±1.49 |
|  | 2 | **6.07±2.32^*T2-5;2-6^** | **6.93±5.38^*T2-5;2-6^** | **4.78±2.27^*T2-5;2-6^** |
|  | 3 | 7.93±3.54 | 7.57±6.68 | 5.46±2.40 |
|  | 4 | 8.08±4.17 | 8.13±8.04 | 4.83±1.27 |
|  | 5 | **9.71±6.09^*T2-5^** | **8.35±9.70^*T2-5^** | **5.15±1.75^*T2-5;2-6^** |
|  | 6 | **11.00±8.80^*T2-6^** | **7.93±6.57^*T2-6^** | **4.81±1.44^*T2-6^** |
| **PCI** | 1 | 16.18±9.42 | 13.78±10.47 | 10.61±5.92 |
|  | 2 | 14.01±5.83 | 14.45±9.21 | 10.08±5.57 |
|  | 3 | 16.57±7.55 | 14.87±11.29 | 11.70±5.57 |
|  | 4 | 17.91±12.81 | 15.06±10.18 | 9.38±4.57 |
|  | 5 | 19.95±13.98 | 15.61±10.68 | 10.10±4.15 |
|  | 6 | 21.44±13.47 | 15.29±10.32 | 9.70±4.96 |

*Abbreviations: WF: persons with multiple sclerosis showing walking fatigability; NWF: persons with multiple sclerosis without walking fatigability; HC: Healthy controls; n: number of participants; PCI: Phase coordination index*

*Bold: significant effect p≤0,05, *T: significant* *main effect of time (post hoc Tukey HSD)*

**Supplementary Table 2.** Spatiotemporal coordination outcomes (mean±standard deviation) per group (HC-WF-NWF) by time (minute 1-6).

| **Outcome** | | **WF (n=13)** | **NWF (n=22)** | **HC (n=13)** |
| --- | --- | --- | --- | --- |
| **Movement amplitude** | Minute 1 | **65.85±21.18 ^G*T1-5;1-6^** | 76.08±18.97 | 73.67±22.02 |
|  | Minute 2 | 63.45±21.88 | 75.34±20.26 | 77.76±25.26 |
|  | Minute 3 | 61.94±21.14 | 74.96±21.51 | 77.91±26.54 |
|  | Minute 4 | 60.49±21.41 | 74.95±21.45 | 78.97±25.98 |
|  | Minute 5 | **58.33±21.26 ^G*T1-5^** | 75.03±23.71 | 78.71±25.34 |
|  | Minute 6 | **57.50±21.50 ^G*T1-6^** | 74.02±23.01 | 79.43±25.28 |
| **Movement frequency** | Minute 1 | 72.57±15.93 | 71.31±12.23 | 82.19±25.51 |
|  | Minute 2 | 73.96±15.70 | 72.56±12.46 | 81.01±23.30 |
|  | Minute 3 | 72.32±15.00 | 72.44±12.27 | 80.77±22.88 |
|  | Minute 4 | 71.67±16.06 | 72.48±12.91 | 81.55±22.29 |
|  | Minute 5 | 71.06±16.43 | 72.84±13.33 | 81.75±21.48 |
|  | Minute 6 | 72.09±17.43 | 72.06±12.89 | 81.95±21.41 |

*Abbreviations: WF: persons with multiple sclerosis showing walking fatigability; NWF: persons with multiple sclerosis without walking fatigability; HC: Healthy controls; n: number*

*of participants*

*Bold: significant effect p≤0,05, ^G*T^: multiple comparison for group*time (post hoc Tukey HSD)*
